# Do all patients that undergo a ‘complete’ secondary cytoreductive surgery for platinum-sensitive recurrent ovarian cancer, benefit from it?

**DOI:** 10.1515/pp-2023-0052

**Published:** 2024-07-08

**Authors:** Aditi Bhatt, Sanket Mehta, Olivier Glehen

**Affiliations:** Department of Surgical Oncology, KD Hospital, Ahmedabad, India; Department of Surgical Oncology, Saifee Hospital, Mumbai, India; Department of Surgical Ocology, Centre Hospitalier Lyon-sud, Lyon, France

**Keywords:** recurrent ovarian cancer, platinum-sensitive recurrence, secondary cytoreductive surgery, patient selection

## Abstract

Following the results of three randomized trials (GOG-213, DESKTOP-III, and SOC-1), secondary cytoreductive surgery (sCRS) is recommended as a therapeutic option for all patients with platinum-sensitive recurrence by the NCCN guidelines and for oligometastatic recurrence by the ESMO-ESGO guidelines. Criteria for predicting a complete gross resection (CGR) were used to select patients for sCRS in all three trials. No trial used surgical prognostic factors like disease sites or disease extent for stratification. The outcomes of sCRS varied in preplanned/post-hoc subgroup analyses. The survival following an incomplete CRS was worse than with systemic chemotherapy alone. Not all patients will benefit similarly from sCRS, even if a CGR is obtained. No trial evaluated the benefit of sCRS in patients receiving poly-ADP ribose polymerase (PARP) inhibitors. While GOG-213 showed no benefit of sCRS when bevacizumab was used, the role of bevacizumab in patients having a CGR was not evaluated. The use of targeted therapies during first-line therapy is increasing, affecting treatment decisions and future clinical trial designs. New trials on sCRS should stratify patients according to surgical prognostic factors; sub-group analyses should be performed only in patients with CGR.

## Highlights

– Selection criteria used in all three randomized trials are predictive of a complete cytoreduction and not the benefit of surgery
– Post-hoc & prespecified subgroup analyses indicate that not all patients undergoing secondary cytoreduction benefit from it
– Post-hoc and sub-group analysis should be performed separately for patients undergoing a complete gross resection
– Impact of incomplete cytoreduction on quality of life and subsequent therapy needs further evaluation
– Future randomized trials should use surgical prognostic factors like disease sites and extent as stratification factors

## Introduction

Despite optimal treatment of advanced epithelial ovarian cancer (EOC), the recurrence rate remains high at more than 70 % [1, 2]. The role of iterative or secondary cytoreductive surgery (sCRS) remains a topic of debate, with one trial showing no survival benefit, another showing a benefit in progression-free survival (PFS) and overall survival (OS) both, and the third one showing a benefit only in PFS (final OS results awaited) [3], [4], [5], [6]. Current guidelines recommend secondary CRS as a treatment option for platinum-sensitive recurrent ovarian cancer [7, 8].

The published clinical trials use selection criteria/scores for a patient selection of sCRS, which are criteria for predicting a complete cytoreduction [4], [5], [6]. Some criteria also have prognostic value. As expected, patients with a favorable prognostic score will live longer, and those with an unfavorable score will have shorter survival rates [6]. Not all patients meeting the selection criteria will derive a similar benefit from sCRS even if a complete cytoreduction/complete gross resection (CGR) is obtained, and some patients may derive no benefit. The decision-making process for surgery is more complex and dependent on several other factors not addressed in these trials. Parallel to the advances in the surgical treatment of ovarian cancer, the fields of systemic chemotherapy (SC) and targeted therapies are also progressing at a rapid pace. Certain patients with BRCA1/2 mutations have a favorable prognosis compared to those without these mutations. They are treated with poly (adenosine diphosphate-ribose) polymerase-PARP inhibitors in the first-line setting, resulting in a significant survival benefit compared to SC alone [9]. This further complicates the selection process for sCRS and the choice of systemic treatment. In this manuscript, we review the three randomized trials published in the current era, evaluating the role of sCRS and focusing on some critical issues regarding patient selection for sCRS. The greater focus of this review is on surgically relevant factors like the experience of centers involved, the number of patients enrolled, factors considered before planning surgery like the number and sites of recurrence, quantification of disease extent, significant morbidity, qualify of life outcomes, time to starting adjuvant chemotherapy. We discuss the need for further trials on the subject and what the design of such a trial could be.

## Overview of the randomized clinical trials

### Outline

#### GOG-213

The first trial to be published was the GOG-213 trial, in which patients with the first platinum-sensitive recurrence from EOC in whom a CGR or complete cytoreduction was deemed possible were included in the surgical objective and randomized to sCRS followed by SC or to SC alone [4]. During the assessment of the chemotherapy objective, surgically amenable patients also underwent randomization (in a 1:1 ratio) to receive either SC alone or SC and bevacizumab.

**Study design:** It was a superiority trial aiming to show the superiority of sCRS and SC over SC alone.

**No of centers and patient enrolment:** 50 centers participated in the trial, of which 50 % enrolled less than 10 patients each. 11 centers enrolled 20 or more patients each (Table 1).

**Table 1:** General information, clinical details, perioperative outcomes and systemic therapies in the GOG-213, DESKTOP-III and SOC-1.

	GOG-213	DESKTOP-III	SOC-1
Number of patients	485	407	357
Recruitment period	Dec 2007 to Jun 2017	Sept 2010 to Dec 2015	July 2012 to June 2019
No of centers	50	30	4
Sites	Multicenter; international	Multicenter; international	Multicenter
Index center/Principal investigator	USA	Germany	China
Primary end-point	OS	OS	PFS
Secondary end-point, s	Investigator assessed PFS	PFS QOL (0,6,12 months) 60 days morbidity	Accumulating treatment-free survival; OS after adjustment of one-way treatment switching (i.e., from the no surgery group to the surgery group); safety; quality of life; validation of the predictive and prognostic value of the iMODEL score; patient compliance; time to first and second subsequent anticancer therapy
No of patients randomized	485	407	357
Stratification factors	1. Prior bevacizumab 2. Platinum-free interval	1. Centre 2. Platinum-free interval	1. Centre2. i-Model score 3. Residual disease at 1st surgery 4. NACT vs. no NACT before 1st surgery
Recurrence site	Extra and intra-abdominal	Extra and intra-abdominal	Extra and intra-abdominal
Cross over to surgery (control violation)	2 %	4 %	6.4 %
Initial stage III/IV	86 %	74.6 %	82 %
Selection criteria	No definite criteria	AGO score	i-Model + PET CT
Serous histology	86 %	85 %	81 %
Median platinum-free interval, months	19.7	19.9	16.1
Complete gross resection	67 %	74.2 %	76.7 %
Surgical procedures			
Peritonectomy	NR	34 %	54 %
Bowel resection	28 %	35.6 %	21 %
Lymphadenectomy	NR	45.5 %	40 %
Hepatic resection	NR	4.7 %	13 %
Extra-abdominal procedures	NR	–	10 %
Major complications	9 %	NR	5 %
Post-operative mortality	0.4 %	0 %	0 %
Surgery in control arm for subsequent recurrence	NR	11 %	36.9 %
Platinum based	100 %	89 %	97.5 %
Bevacizumab (1st line)	12.2 % in the surgery and 10.4 % in the no surgery groups	NR	1 %
PARP inhibitors (1st line)	NR	NR	7 % in the surgery and 4 % in the no-surgery groups
Bevacizumab (2nd line)	84 %	23 %	1 %
PARP inhibitors (2nd line)	NR	<5 %	10 %

OS, overall survival; PFS, progression-free survival; NACT, neoadjuvant chemotherapy.

#### DESKTOP-III

In the DESKTOP-III trial, patients with clinical or radiological evidence of recurrent disease meeting the AGO (Arbeitsgemeinschaft Gynäkologische Onkologie) criteria were randomized (1:1) to undergo sCRS followed by SC or receive SC alone [5]. Anticipating a 2-year OS of 66 % in the surgery group and 55 % in the no-surgery group, the hazard ratio for death was set at 0.70 (Table 1).

**Study design:** Superiority trial.

**Participating centers:** There were 30 centers from Europe, China, and South Korea. Information on the number of patients enrolled by each center is not available.

#### SOC-1

In the SOC-1 trial, patients with the first platinum-sensitive recurrence from EOC diagnosed by the RECIST 1.1 criteria were randomized to sCRS followed by SC or SC alone [6]. The hazard ratio for PFS was set at 0.61, anticipating an 18 % increase (22–40 %) in the PFS and for OS at 0.68 (corresponding to a rise from 60 to 70.5 % in the 3-year OS). 172/182 had per protocol treatment in the sCRS + SC arm, and 158/175 had per protocol treatment in the SC arm.

**Study design:** Superiority trial.

**Participating centers:** Four high-volume centers. The index centre recruited 171 patients, with the other centres contributing 94, 84, and 25 patients respectively.

### Selection criteria for secondary CRS and disease sites and surgical procedures

Apart from the general selection criteria for any surgery and platinum sensitivity, criteria for predicting a CGR were used in two of the three trials.

#### GOG-213

There were no predefined criteria for predicting a CGR at sCRS in the GOG-213 trial. Patients judged eligible for a complete cytoreduction or a CGR by the investigator were randomized to surgery or no surgery [4]. The disease sites in patients undergoing sCRS included intra-abdominal and extra-abdominal (10 %) sites. 36 % of the patients had disease at only one site and 46 % at more than two sites of disease. Liver involvement included both parenchymal liver metastases and liver surface/capsular involvement. 2 % of the patients crossed over to the surgery group from the no-surgery group.

#### DESKTOP-III

The DESKTOP I and II trials defined and validated the predictive score (the AGO score) [10], [11], [12]. Only patients with a positive AGO score were included in the trial.

Patients with extra-abdominal recurrence were also included, although the percentage was tiny (1.5 % in the surgical arm). The most typical disease site was the pelvis, the upper abdomen, and the retroperitoneal lymph nodes [5]. The trial does not provide information on the number of sites in each patient [5].

#### SOC-1

Patients likely to have a complete resection using the iMODEL score combined with PET-CT imaging were enrolled in the SOC-1 trial [6]. Four subsequent protocol modifications were made, one of which was to include PFS as a primary end-point because of an unexpectedly high rate of one-way treatment, switching from no surgery to surgery [6, 13].

The disease sites included both extra-abdominal and intra-abdominal sites. The patients were divided into three groups according to the number of recurrent lesions (1–3; 4–19; ≥20).

### Perioperative outcomes

#### GOG-213

A CGR was achieved in 67 % (of 224 patients with secondary CRS). Bowel resection was performed in 28 %, and a stoma was created in 2 % (Table 1). Major complications within 30 days occurred in 9 % (n=20) and 0.4 % (n=1) patients who died following surgery [4]. The median time to start adjuvant chemotherapy was 40 days in patients undergoing surgery (IQR 28–51 days) and seven days (IQR 4–11 days) in the chemotherapy-alone group. The details of specific complications are not available.

#### DESKTOP-III

A CGR was achieved in 75.5 %, residual disease measuring<1 cm was observed in 9.9 %, and >1 cm in 10.9 % [5]. A temporary ostomy was created in 3.7 % and permanent in 4.2 %. The absolute morbidity figures are not provided, but the re-surgery rate was 3.7 %, and no patient died within 30 days of sCRS (Table 1). Infections requiring antibiotics were reported in 19.4 %. Results of quality-of-life analyses did not show any between-group differences concerning global health status, quality of life, or any functional subscale at baseline, visit 1 (at six months), or visit 2 (at 12 months).

#### SOC-1

The median time between randomization and sCRS was seven days (IQR (inter-quartile range) 5–8). A CGR was obtained in 76.7 % [6]. In patients with an iMODEL score of more than 4.7 assigned to surgery, 11/18 (61 %) had a CGR, and 121/154 (79 %) patients with an iMODEL score less than 4.7 assigned to surgery had a CGR.

This trial reported details of the complications, with pleural effusion (2 %) being the most common post-surgical complication.

None of the 38 patients with bowel anastomosis developed an intestinal fistula (Table 1).

The median time between sCRS and SC initiation was 16 days (IQR 13–21) in the surgery group. The median time between randomization and SC was two days (1–4) in the no-surgery group. The prespecified patient-reported outcomes based on EORTC QLQ-C30 global health status and FACT-O TOI score did not differ between the two groups.

### Systemic therapies

#### GOG-213

The paclitaxel-platinum doublet was used in 69 % of the patients, and gemcitabine and carboplatin were used in the remaining patients [4]. Bevacizumab was used in 84 % (408/485). It is unclear whether patients in the surgical arm were randomized to bevacizumab or no-bevacizumab or if it was based on the investigator’s choice. However, the proportion of patients receiving bevacizumab was similar in both arms. No information is available on the number of patients completing all the prescribed cycles of chemotherapy and bevacizumab. Information on the use of PARP inhibitors is not available.

#### DESKTOP-III

76.7 % of the patients in the surgery group and 79.6 % in the no-surgery group received at least five cycles of SC [5]. A total of 94 patients (23 %; 47 in each group) received bevacizumab as part of second-line therapy, and only a few patients (<5 %) received a PARP inhibitor during the trial (8 in the surgery group and 12 in the no-surgery group).

#### SOC-1

A total of 176 (97 %) of 182 in the surgery group and 168 (96 %) of 175 in the no-surgery group received second-line SC [6]. 35 (20 %) of 176 patients in the surgery group and 44 (26 %) of 168 patients in the no surgery group received less than six cycles of SC due to disease progression, a decrease in treatment decided on by the patients or their treating clinician, toxicity, or subsequent maintenance therapy as decided by the investigators. Overall, 11 % in the no-surgery group (1 % during second-line therapy) and 8 % in the surgery group (1 % during second-line therapy) received maintenance bevacizumab. 16 % in the surgery group (9 % during second-line therapy) and 15 % in the no-surgery group (11 % during second-line therapy) received PARP inhibitors.

### Survival outcomes

#### GOG-213

The hazard ratio (HR) for death (surgery vs. no surgery) was 1.29 (Table 2) [4]. The HR for disease progression or death (surgery vs. no surgery) was 0.82 (95 % CI, 0.66 to 1.01; PFS, 18.9 and 16.2 months, respectively). There was a significant benefit in both OS (HR 0.61, 95 % CI 0.40–0.93; median 56.0 vs. 37.8 months) and PFS (HR 0.51, 95 % CI 0.36–0.71; median 22.4 vs. 13.1 months) in patients who had a complete gross resection (CGR) compared to those that did not. The HR for death was 1.03 (95 % CI 0.74–1.46); the median was 56.0 months for CGR vs. 64.7 months for SC alone. For disease progression, the HR was 0.62 (95 % CI 0.48–0.80) median of 22.4 vs. 16.2 months (CGR vs. SC alone). It is unclear why patients undergoing a CGR had a superior PFS and inferior OS compared to those receiving SC alone, and the authors have not addressed this.

**Table 2:** Survival outcomes the GOG-213, DESKTOP-III and SOC-1 trials.

	GOG-213	DESKTOP-III	SOC-1	
Median OS (secondary CRS) (months)	50.6	53.7	58.1	
Median OS (no surgery) (months)	64.7	46.0	53.9	
HR (95 % CI)	1.28 (0.97–1.72); p-NS	0.75 (0.58–0.96); p=0.004	0.82 (0.57–1.19); p-NS	

Pre-defined subgroup analyses		HR (95 % CI)		p-Value

Platinum-free interval				
6-12 months	0.92 (0.54–1.56)	0.59 (0.37–0.94)		
>12 months	1.43 (1.03–2.00)	0.83 (0.62–1.11)		
Prior bevacizumab				
Yes	1.37 (0.70–2.69)	0.75 (0.39–1.43)^a^		
No	1.27 (0.93–1.72)	0.74 (0.56–0.97)^a^		
Centre				
Fudan			0.27 (0.15–0.47)	0.05
Zhenjiang	–	–	0.78 (0.48–1.26)	
Sun Yat			0.99 (0.39–2.63)	
Zhongshan			0.60 (0.41–0.89)	
i-model score				
<4.7	–	–	0.62 (0.45–0.86)	0.973
4.7			0.39 (0.23–0.66)	
>4.7			0.71 (0.37–1.37)	
Residual disease at 1st surgery				
absent	–	–	0.69 (0.47–1.02)	0.204
present			0.49 (0.35–0.69)	
NACT before 1st surgery				
No	–	–	0.48 (0.36–0.63)	
Yes			1.20 (0.63–2.28)	0.008

^a^post-hoc analysis. OS, overall survival; CRS, cytoreductive surgery; HR, hazard ratio; NACT, neoadjuvant chemotherapy.

While a detriment in OS was observed among those undergoing surgery but not receiving bevacizumab (HR: 2.26, 95 % CI: 1.31–3.88), the impact of bevacizumab in patients with a CGR was not studied.

#### DESKTOP-III

The HR for death favored surgery over no surgery (HR: 0.75; 95 % CI, 0.59 to 0.96; p=0.02) [5]. The median PFS was 18.4 months (95 % CI, 15.7 to 20.8) in the surgery group and 14.0 months (95 % CI, 12.7 to 15.4) in the no-surgery group (HR for progression or death, 0.66; 95 % CI, 0.54 to 0.82). In patients with complete resection, the median OS was 61.9 vs. 27.7 months in those with incomplete resections. The median OS in patients not achieving a CGR was 20 months less than those not undergoing surgery and 32 months less than those achieving a CGR. Thus, an incomplete cytoreduction had a significant adverse prognostic impact on the OS. However, it may be argued that incomplete surgery did not induce this, but it was a consequence of aggressive disease biology. In patients who did not receive bevacizumab, the hazard ratio for OS was 0.67, favoring sCRS, and for PFS, 0.61, also favoring sCRS. Due to the small numbers, the survival analyses were not performed in patients receiving bevacizumab.

#### SOC-1

The median PFS was 17.4 months in the surgery group and 11.9 months in the no-surgery group (HR 0.58, 95 % CI 0.45–0.74; p<0.0001) [6]. The stratified Cox proportional hazards model showed a similar result (HR 0.56, 95 % CI 0.43–0.72; p<0.0001). The PFS benefit was seen in patients with a CGR (HR-0.50 for CGR vs. no surgery) but not in patients with gross residual disease (HR-0.91 for gross residual disease vs. no surgery).

Median time to first subsequent anticancer therapy was 18.1 months with surgery vs. 13.6 months with no surgery (HR 0.59, 95 % CI 0.46–0.76); median time to second subsequent anticancer therapy was 33.5 months following surgery group vs. 28.1 months with no surgery (HR 0.69, 0.51–0.94).

The data for OS are not mature, but in the interim analysis (after 105 deaths were recorded), the median OS was 58.1 months (95 % CI not estimable to not estimable) in the surgery group and 53.9 months (42.2–65.5) in the no surgery group (HR 0.82, 95 % CI 0.57–1.19).

### Pre-planned and post-hoc analyses of prognostic factors

#### GOG-213

The stratification factors for the surgical objective were the platinum-free interval and the prior use of bevacizumab [4]. Patients with a PFI>12 months (HR-1.43) seemed to have a more significant benefit in OS from systemic chemotherapy alone compared to those with a PFI 6–12 months (HR-0.92) (Table 2). Patients with liver and extra-abdominal disease (HR for OS 3.50) derived the most negligible benefit from surgery, followed by other sites (non-liver, non-extra-abdominal; HR-1.36), while HR for OS for liver metastases was 0.92. There was a variation according to the country, with patients undergoing surgery in Japan (n=31) having an HR for death of 0.33, compared to 0.97 in South Korea (n=50) and 1.43 in the United States.

#### DESKTOP-III

In the DESKTOP-III trial, patients were stratified according to the center and the platinum-free interval [5]. The HR for OS was 0.59 for recurrence within 6–12 months and 0.83 for patients developing recurrence after 12 months, indicating that patients who recurred early benefitted more from surgery (Table 2). Patients aged >65 years had a more favorable HR compared to those aged less than 65 years (0.62 and 0.84, respectively), and patients with stages III and IV at diagnosis had a more favorable HR compared to stages I and II at diagnosis (0.72 and 0.87 respectively). These results are contrary to what would be expected. There is no information on the impact of disease sites on survival.

#### SOC-1

In the preplanned PFS analysis, patients who had an i-Model score of 4.7 seemed to derive the maximum benefit (HR-0.39) compared to those with a score of <4.7 (HR-0.62) or >4.7 (HR-0.72) [6]. Patients who did not receive NACT benefitted from surgery (HR-0.48), while those who received NACT before the first surgery did not benefit from secondary CRS (HR-1.20). The index center (Fudan Hospital) had the most favorable outcomes (HR-0.27 favoring surgery), whereas the results at the other centers were not similar (HR-0.60, 0.78, and 0.99 at the three different centers). Patients with gross residual disease (HR-0.49) at the first surgery had better HR-favoring surgery than those without residual disease (HR-0.69).

## Do all patients undergo a CGR benefit from sCRS?

The outcomes of patients undergoing CGR were not analyzed separately in all three trials. It is impossible to answer this question based on the results of these trials. This can only be assumed to be accurate based on the post-hoc analyses in which patients recurring early, those older than 65, and those with stage III/IV disease at diagnosis derived more significant benefit from sCRS than their counterparts.

**Figure 1:**
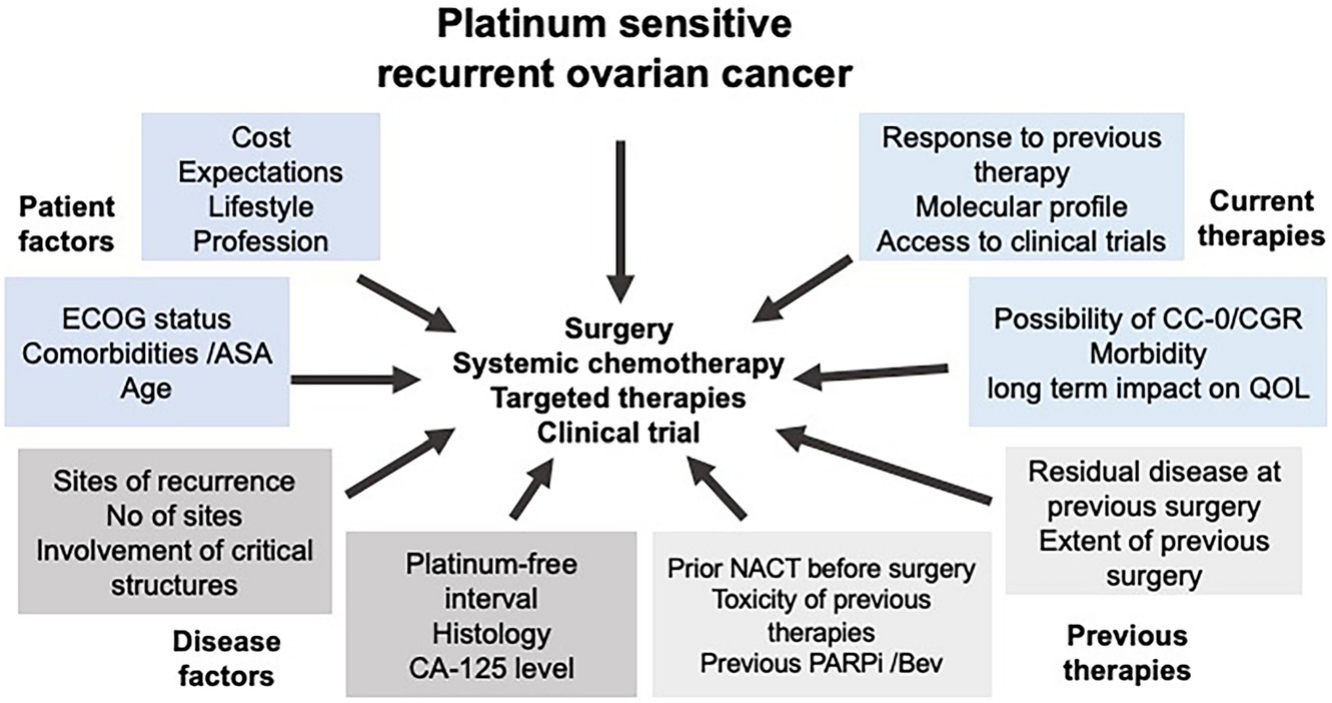
Factors to be considered in patient section for secondary cytoreductive surger. ASA, American society of anesthesiologists score; ECOG, eastern co-operative oncology group status; CC-0, completeness of cytoreduction score −0 or no macroscopic residual disease; CGR, complete gross resection; NACT, neoadjuvant chemotherapy; QOL, quality of life.

It is clear from the DESKTOP 3 (OS difference of 3 years between incomplete surgery and CGR) and the GOG-213 (OS difference of 1.5 years between the two groups) that the survival with an incomplete sCRS is inferior to that achieved with SC alone [4, 5]. Thus, one of the main goals of sCRS should be to obtain a CC-0 or complete gross resection. And since one in four patients will have an incomplete cytoreduction, more careful patient selection and counseling are needed. However, several other factors must be considered for patient selection. The patient-related factors would include performance status, age, and comorbidities. The disease-specific factors include the site or sites of the disease, the extent of the disease, the histological subtype, the molecular profile, and the platinum-free interval. The treatment-related factors would include the extent of surgery that would be required to achieve a CGR and its impact on the quality of life, the previous systemic therapies administered, including bevacizumab and PARPi and their toxicities, and the cost and availability of different treatments (Figure 1). While the prognostic impact of some of these factors has been demonstrated, that of several factors has not been proven; some, like the cost of therapy, are logistic factors.

Several validated and non-validated scores are used to select patients for sCRS, including one or more of these prognostic factors [14]. The primary purpose of these scores is to predict the possibility of a complete cytoreduction or CGR. These predictive scores and the other prognostic factors not included in these scores are discussed below. Two randomized trials did not show a negative impact of sCRS on the QOL, barring the immediate postoperative period. However, there is no information on the subgroup with no CGR [5, 6].

### Predictive scores for a complete gross resection

The Memorial Sloan-Kettering (MSK) score was the first to be developed (Table 3) [15]. The validated and most widely used score is the AGO score (Table 3), and using the AGO criteria, a CGR was achieved at 76 % in the validation study, but the negative predictive value was 38 % and specificity only 53 % [14]. In one validation study, 63 % with a negative AGO score had a CGR [11]. In a study that used the Tian model (i-model) (Table 3), patients with a low risk of incomplete cytoreduction had a score of 0–4.7, a CGR rate of 53.4 %, and patients with a CGR had a median OS of 63 months, whereas, those with a high risk had a score of >4.7, a CGR rate of 20.1 % and a median OS of 40 months in patients with a CGR [16]. External validation of the Tian model showed sensitivity and specificity values of 83.3 and 57.6 %, respectively [17]. Whereas this score stratifies patients into different prognostic sub-groups, it is unclear if the low-risk group benefits more from surgery than the high-risk group or vice versa. In the SOC-1 trial, patients with an i-model score of 4.7 seemed to benefit the most from surgery (Table 2) [6]. One retrospective study showed a good concordance between the MSK and Tian scores (both accurately predicted a CGR in more than 80 %), while the AGO score was accurate only in 49 % [18]. The AGO score and Tian model have been validated, and the MSK score has not been validated in randomized trials [5, 6]. It has been proposed that the AGO score should be applied first, followed by the MSK score to select patients for surgery [18]. The Tian score or i-model criteria may be used for further stratification in patients with an intermediate MSK score. However, as with the Tian score, none of these scores can point out the magnitude of the benefit from sCRS.

**Table 3:** AGO score, MSK criteria and the tian model.

SCORE and its description							Prognostic value	Predictive value
The AGO score								

**Predictive parameters of CGR in platinum-sensitive recurrent ovarian cancer (AGO score)**							**No**	For CGR Yes
Good performance status (ECOG 0)								
No residual disease after primary surgery (or, alternatively, FIGO I/II)								For benefit from sCRS No
Absence of ascites on preoperative imaging (<500 cc)								

The MSK criteria								

**Disease-free interval**	**Single site**		**Multiple sites: No carcinomatosis**			**Carcinomatosis^a^**	**Yes**	For CGR Yes
6–12 months	Offer sCRS		Consider sCRS			No sCRS		
12–30 months	Offer sCRS		Offer sCRS			Consider sCRS		For benefit from sCRS No
>30 months	Offer sCRS		Offer sCRS			Offer sCRS	

The tian model (i-model criteria)								

**Impact factors**	**Scoring**						**Yes**	For CGR Yes
	**0**	**0.8**	**1.5**	**1.8**	**2.4**	**3.0**		
Figo stage	I/II	III/IV						
RD after primary surgery	0		>0					
PFI, months	>16				<16			For benefit from sCRS No
ECOG performance status	0–1				2–3			
CA125 at recurrence, U/ml	<105			>105				
Ascites at recurrence	Absent					Present		

CGR, Complete gross resection; ECOG, Eastern co-operative oncology group; MSK, Memorial Sloak Kettering; sCRS, secondary cytoreductive surgery; ^a^Carcinomatosis is defined as 20 or more tumor nodules during surgery; FIGO, International Federation of Gynecology and Obstetrics; RD, residual disease; PFI, progression-free interval.

### Other criteria for predicting a CGR

For any CRS procedure, surgical decision-making is based on the extent of the disease and the involvement of critical anatomical structures that would preclude a complete cytoreduction or have a detrimental impact on the long-term quality of life. For example, involvement of the porta hepatis in a patient who has had prior surgery in that region could preclude a CGR even if the disease is limited.

Involvement of the bladder trigone would necessitate a cystectomy with or without exenteration, which may not be advocated, especially if there is a peritoneal disease [19]. Another factor that needs to be considered in cases with disease in the small bowel is the length of the remaining small bowel and its impact on the quality of life. The evaluation of disease extent is based on cross-sectional imaging studies with or without the addition of a staging laparoscopy. All three scores predict resectability as a function of other prognostic factors and not based on objective radiological features for resectability. The only radiological findings included are the presence of ascites and multiple disease sites [15, 16]. It may be assumed that imaging findings were considered in decision-making. In a multi-institutional study on iterative CRS for colorectal peritoneal metastases, a CC-0/1 resection was achieved in 83.5 % of the patients [20].

Future efforts need to focus on reducing the proportion of patients having an incomplete resection, and scores and selection criteria should incorporate imaging features and/or sites of disease.

### Should patients not be offered sCRS outside a clinical trial in light of current evidence?

Given that one out of four patients will have an incomplete resection and the possibility of a poorer survival compared to SC alone, should sCRS be offered to patients outside clinical trials?

The NCCN (National Comprehensive Cancer Network) guidelines recommend sCRS as a therapeutic option for patients with platinum-sensitive recurrence if the AGO criteria are fulfilled and a CGR can be achieved [7]. The ESMO (European Society of Medical Oncology)-ESGO (European Society of Gynaecologic Oncology) guidelines recommend sCRS for oligometastatic platinum-sensitive first recurrence [8]. The rate of CGR in clinical trials should not be the benchmark, but the insights gained from these trials should be used for better patient selection, limiting sCRS to only those with a high likelihood of CGR. Other factors that should be considered are discussed below.

### Sites of disease and disease extent

None of the three trials uses either the sites of disease or the disease extent as a stratification factor, and information on the impact of this critical prognostic factor is missing. All three included patients with both intra-abdominal and extra-abdominal disease were included. According to the number of lesions, the hazard ratios for PFS in the SOC-1 trial were 0.59 for 1–3 lesions, 0.52 for 4–19 lesions, and 0.57 for >19 lesions. It has not been specified how the number of lesions was considered-three lesions confined to the pelvis cannot be compared to three lesions in different parts of the abdominal cavity (e.g., in the pelvis, mid and upper abdomen) [6]. This information on the disease sites is crucial from the morbidity point of view: one 3 cm nodule on the lesser curve of the stomach cannot be compared to a solitary 3 cm nodule in the pelvis or the abdominal wall. One extrapolation from the trials could be that when the margin of benefit is small, and the anticipated morbidity is high, systemic therapy alone could be a reasonable option. Peritoneal metastases, intra-abdominal lymph nodes, pleural metastases, parenchymal liver metastases, and mediastinal nodes are the most common sites of recurrence/progression that are likely to have different prognosis but are all considered together. In the GOG-213, the worst hazard ratios for surgery were for intra-abdominal disease as compared to liver metastases and extra-abdominal sites of disease [4]. sCRS is a non-curative surgery for most patients, potentially prolonging survival and preserving the quality of life. In patients with limited but chemo-sensitive disease and a long platinum-free interval, can SC induce a complete response and produce the same survival as sCRS followed by SC? If the Gompertzian kinetics is considered, chemotherapy should be more effective on smaller or limited tumor burden [21]. In the GOG-213, the median OS with systemic treatment alone was more than 64 months [4]. Thus, some patients experience prolonged survival with SC and targeted therapies alone. These are crucial questions for decision-making by multidisciplinary teams and for conveying accurate prognosis and treatment options to patients.

### Isolated recurrence

In one of the first studies in the modern era on sCRS, patients with a single site or isolated recurrence had significantly better OS after sCRS (60 m vs. 42 months for multi-site disease vs. 28 months for carcinomatosis; p<0.001) [15]. This study is nearly 20 years old and included patients treated before 2001, during which the techniques of CRS were being developed. In-field recurrence after peritonectomy could differ from recurrence when no peritonectomy has been performed for the reasons discussed here. First, the peritoneum is the first line of defense, which prevents retroperitoneal tumor extension when not breached [22]. Prior surgery breaks this barrier, and following a peritonectomy, recurrent tumors will infiltrate a varying extent of the retroperitoneum or the underlying parietal and visceral structures. Due to this, even if a CC-0 resection or CGR is performed, it will be an R1 resection as there is a high probability of microscopic residual disease at the resection site. Second, diagnosing recurrence on imaging may be more challenging than exploring the abdomen thoroughly after a previous extensive CRS. Staging laparoscopy may not be able to evaluate all regions [23]. Thus, there could be more disease than is visible, and the recurrence may not be truly isolated. A meticulous evaluation of the disease extent should be performed. MRI peritoneum may be a valuable tool in this regard [24].

### Is there a role of sCRS in patients receiving bevacizumab?

In the GOG trial, bevacizumab, in addition to SC, produced a similar or superior survival when compared to sCRS with SC with/without bevacizumab. However, the role of bevacizumab was not considered in patients undergoing a CGR. The results of GOG-213 cannot be interpreted as having no role in sCRS, though the median OS achieved with systemic therapies alone is remarkable [25]. Current treatment guidelines recommend the use of bevacizumab during first-line therapy, which has led to the widespread use of bevacizumab in patients with advanced EOC. Approximately 50 % of patients in high-income countries receive first-line maintenance bevacizumab [26, 27]. In the GOG-213, bevacizumab was used only in 11 % during first-line treatment. Hence, it cannot be assumed that if bevacizumab is reintroduced, it will provide the same survival benefit as when used for the first time [28]. Bevacizumab was used in only 23 % of the patients in the DESKTOP-III trial, and therefore, the benefit of sCRS in patients receiving the drug could not be elucidated [5]. In GOG-213, non-serous and non-clear subtypes benefited from secondary CRS [HR-0.59 (95 % CI 0.21–1.69)] despite using bevacizumab [4]. More evidence is needed to identify which patients could be treated with SC alone when bevacizumab is used and which patients should have sCRS in addition to bevacizumab and SC. The side effects of systemic treatment, the cost of treatment, and the long-term impact on quality of life should be considered.

### Is there a role of sCRS in the era of PARP inhibitors?

PARP inhibitors are now recommended as second-line therapy irrespective of the BRCA mutation status [9]. In all three trials, they were used in <20 % of the patients, and therefore, no conclusions can be derived from these trials on the benefit of sCRS in patients receiving PARPi. In a retrospective study of 126 patients treated between 2005 and 2016, there was no benefit in post-recurrence survival with secondary CRS in patients with BRCA1/BRCA2 mutated tumors, while a benefit was in BRCA1/2 wild-type tumors [29]. In a subsequent propensity score matched study, the same investigators showed that the median time to first subsequent therapy (TFST) was significantly longer in the patients who had sCRS in addition to SC and olaparib compared to systemic therapies alone (42 vs. 16 months; p=0.05) and patients undergoing sCRS also had a more prolonged 3-year post recurrence survival (79 vs. 42 %; p=0.02) [30].

Conversely, another retrospective study of 127 patients showed a benefit of sCRS irrespective of the BRCA1/BRCA2 mutation status. However, only 4 % of the patients received maintenance olaparib [31]. More evidence is needed on the role of sCRS in patients with BRCA mutation or homologous repair deficiency, those receiving PARP inhibitors, or bevacizumab as second-line maintenance therapy. Current ESMO-ESGO guidelines still recommend sCRS for oligometastatic, completely resectable, recurrent disease, irrespective of targeted therapies. The role of sCRS in PARP inhibitors (niraparib) is being evaluated in a phase II multi-centre RCT (SGOG SOC-3 Study, NCT03983226).

With more and more patients receiving PARPi during first-line therapy, the role of sCRS in patients with/without BRCA mutations will need careful evaluation [32, 33].

Two recent publications showed that patients having oligometastatic progression on PARP inhibitor maintenance therapy, either during first-line treatment or for recurrence, benefit from locoregional treatment, including surgery [34, 35].

**Figure 2:**
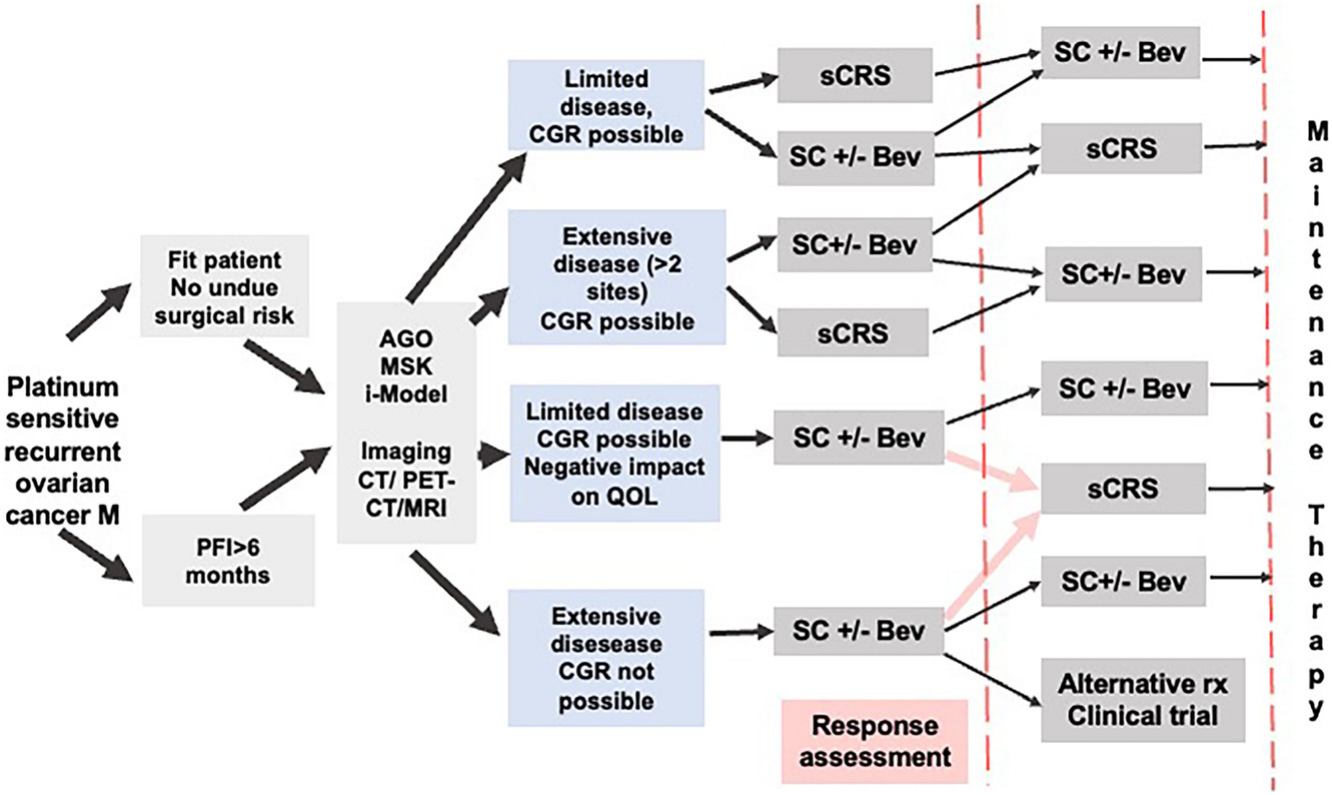
Algorithm for selection of patients for secondary cytoreductive surgery and systemic therapies. PFI, platinum-free interval; CGR, complete gross resection; QOL, quality of life; sCRS, secondary cytoreductive surgery; SC, ystemic chemotherapy; Bev, Bevacizumab.

The indications and timing of sCRS for recurrent ovarian cancer are evolving in patients who have received first-line maintenance and will receive second-line maintenance therapy Figure 2.

### Do all patients undergoing sCRS need second-line SC?

SC is the current standard of care for the treatment of recurrent EOC [36, 37]. sCRS is recommended for selected patients. Could SC be omitted or deescalated in patients with BRCA mutated tumors undergoing sCRS? Future trials should answer this question. Current ESMO-ESGO guidelines and NCCN guidelines do not recommend giving NACT to patients in whom sCRS is planned. There are two situations:

– The first situation is when the recurrent disease is unresectable, and SC is used to reduce the disease burden and enable resection. sCRS may be of limited benefit since these patients generally have ascites and extensive peritoneal involvement. Intraperitoneal chemotherapy could have a role in these difficult-to-treat patients, which should be investigated in future studies [38].
– The second situation is when SC is administered first, even when the disease can be removed surgically. SC may allow testing of the disease biology and responsiveness to SC. Complete responders could be treated with consolidation or maintenance therapy, especially if significant morbidity is expected. A schema for selecting patients for sCRS and SC is outlined in Figure 2. A recent randomized trial showed the benefit of adding hyperthermic intraperitoneal chemotherapy to sCRS. Though the entire publication is awaited, it would be interesting to see where HIPEC fits in with the systemic and maintenance therapies [39].

## Future perspective

**Figure 3:**
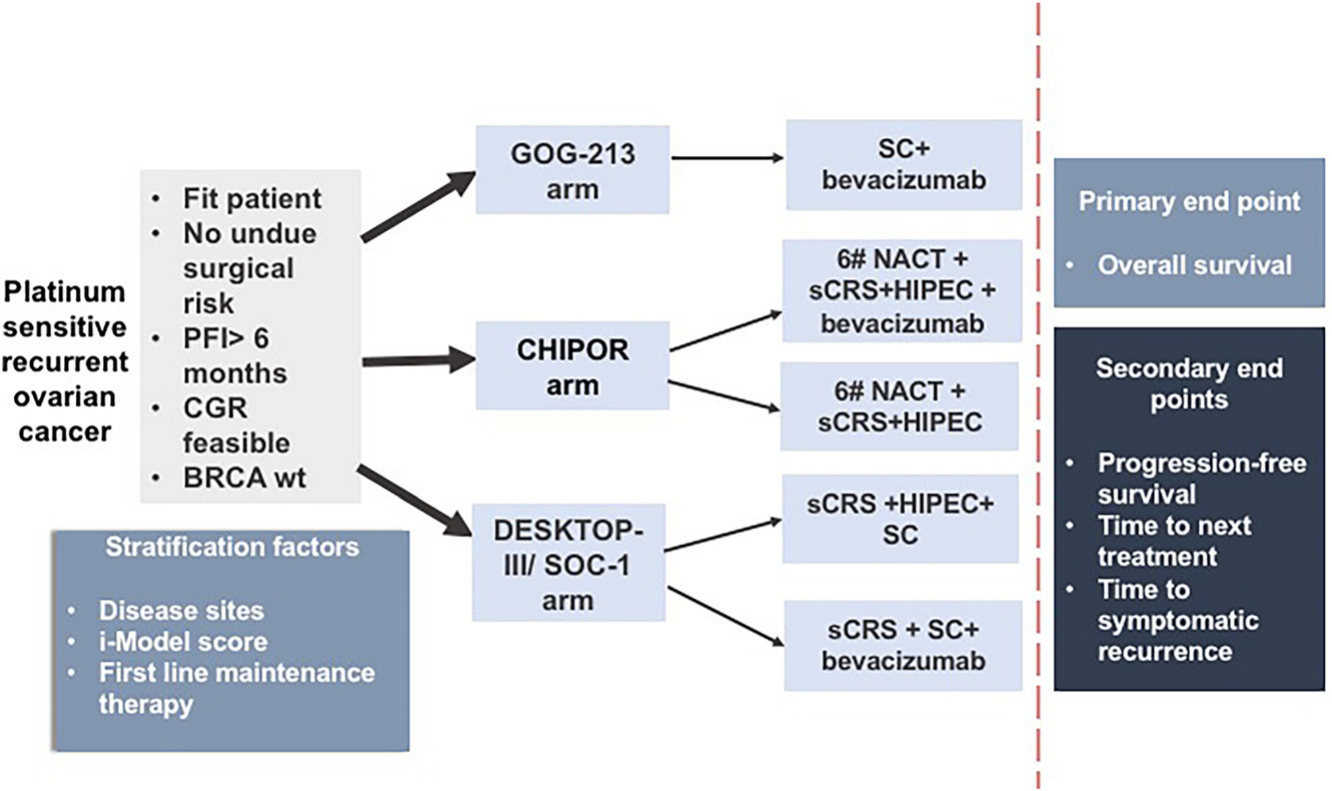
Proposed design for a randomized trial evaluating the role of secondary cytoreductive surgery in the setting of other regional and systemic therapies in patients with BRCA wild type tumours. PFI, platinum-free interval; wt, wild type; CGR, complete gross resection; sCRS, secondary cytoreductive surgery; NACT,neoadjuvant chemotherapy; SC,systemic chemotherapy;

There was a survival benefit following sCRS only with complete CRS/CGR in all three trials. Future efforts should be focused on minimizing the number of patients with incomplete cytoreduction. It is unclear if the poorer survival in patients undergoing incomplete cytoreduction is due to aggressive disease biology or detrimental effects on quality of life and the ability to undergo subsequent therapies. This needs further evaluation. These trials do not provide adequate information on surgical prognostic factors like the sites and extent of disease. The benefit of sCRS remains to be demonstrated in patients receiving targeted therapies and will be influenced by the use of these treatments during first-line therapy. Ongoing and future trials represent an opportunity to evaluate the role of sCRS and stratify patients according to surgical prognostic factors. In all three trials, sub-group analyses are performed on the whole cohort of patients undergoing surgery-to demonstrate the benefit in different sub-groups. These analyses should be performed only in patients with a CGR or after adjustment for this significant confounding factor. This information will be crucial in bringing objectivity and standardizing the indications for sCRS. A hypothetical design for a randomized trial evaluating the role of sCRS in other regional and systemic therapies in patients with BRCA wild-type tumors is presented in Figure 3.

Given the rapidly expanding number of systemic therapies showing a survival benefit, there may be more than one therapeutic option for every clinical situation, thus making it difficult to standardize indication for sCRS. The indications for sCRS could be narrowed down, but at the same time, new indications for sCRS or tertiary CRS are likely to emerge-e.g., oligometastatic progression on maintenance therapy.

## Conclusions

Though secondary cytoreductive surgery is currently recommended as a treatment option for selected patients with recurrent platinum-sensitive ovarian cancer, its role in the setting of bevacizumab or PARPi used as first-line maintenance or second-line maintenance therapy needs further evaluation. From the three trials published so far, it is unclear if all patients undergoing a CGR benefit from sCRS. Selection criteria for sCRS need to be refined and standardized. With the increase in the use of bevacizumab and PARPi as first-line maintenance therapy, new clinical trials are required to define the role and indications of sCRS and provide crucial information on surgical prognostic factors by stratifying patients according to these factors. Sub-group analysis should be performed only in patients with a CGR or after accounting for this significant confounding factor.
